# Herbal essential oils improve growth, antioxidant response, and gene expression in Nile Tilapia fingerlings

**DOI:** 10.3389/fvets.2025.1620632

**Published:** 2025-09-10

**Authors:** Malik Khalafalaa, Shaimaa M. Shehab, Mohamed H. Aboraya, Asem A. Amer, Foad Farrag, Mohamed F. Abdelghany, Badriyah S. Alotaibi, Mohamed Abdelmegeid, Mustafa Shukry, Ahmed A. Elolimy

**Affiliations:** ^1^Department of Fish Nutrition, Faculty of Aquatic and Fisheries Sciences, Kafrelsheikh University, Kafrelsheikh, Egypt; ^2^College of Fisheries and Life Science, Shanghai Ocean University, Shanghai, China; ^3^Department of Aquaculture, Faculty of Aquatic and Fisheries Sciences, Kafrelsheikh University, Kafrelsheikh, Egypt; ^4^Department of Fish Nutrition and Feed Technology, Central Laboratory for Aquaculture Research, Agricultural Research Center, Abbassa, Abo-Hammad, Egypt; ^5^Department of Anatomy and Embryology, Faculty of Veterinary Medicine, Kafrelsheikh University, Kafrelsheikh, Egypt; ^6^Department of Fish Production, Faculty of Agriculture, Al-Azhar University, Cairo, Egypt; ^7^Department of Pharmaceutical Sciences, College of Pharmacy, Princess Nourah bint Abdulrahman University, Riyadh, Saudi Arabia; ^8^College of Veterinary Medicine, University of Al Dhaid, Sharjah, United Arab Emirates; ^9^Department of Animal Medicine, Faculty of Veterinary Medicine, Kafrelsheikh University, Kafrelsheikh, Egypt; ^10^Department of Physiology, Faculty of Veterinary Medicine, Kafrelsheikh University, Kafrelsheikh, Egypt; ^11^Department of Integrative Agriculture, College of Agriculture and Veterinary Medicine, United Arab Emirates University, Abu Dhabi, United Arab Emirates

**Keywords:** Nile tilapia, herbal essential oils, growth performance, immune-related genes, antioxidant enzymes, intestinal morphology

## Abstract

**Introduction:**

The increasing global demand for sustainable aquaculture practices has prompted the search for natural and effective alternatives to synthetic feed additives. Herbal essential oils (HEOs) have emerged as promising candidates due to their bioactive properties that support growth, health, and immunity in fish.

**Methods:**

This study evaluated the effects of dietary supplementation with blended HEOs—comprising carvacrol, oregano oil, 1,8-cineole, thymol, *α*-pinene, *β*-pinene, limonene, and propylene glycol—on growth performance, hematological indices, antioxidant status, immune response, intestinal morphology, and gene expression in Nile tilapia (*Oreochromis niloticus*) fingerlings. Over a 72-day trial, fish were fed diets with 0 (control), 30, 60, 120, and 240 mL/kg of HEOs.

**Results and discussion:**

The 30 and 60 mL/kg groups showed significantly improved final body weight, weight gain, specific growth rate, and feed conversion ratio (*p* < 0.05). Hematological parameters increased, while serum cholesterol and triglyceride levels decreased. Enhanced lysozyme activity, phagocytic rate, IgM concentration, and antioxidant enzymes (SOD and CAT) were observed in the 30 and 60 mL/kg groups. Additionally, these doses significantly upregulated the expression of growth- and immunity-related genes (GHr, IGF-I, IL-1β, TNF-*α*, ZO-1, and occludin) while downregulating HSP70, indicating improved stress resilience. Histological analysis revealed increased villi height and surface area in the intestine, suggesting better nutrient absorption. These findings demonstrate that dietary supplementation with 30–60 mL/kg of HEOs can enhance physiological and immunological health, offering a natural strategy to improve Nile tilapia aquaculture productivity.

## Graphical abstract

**Figure:**
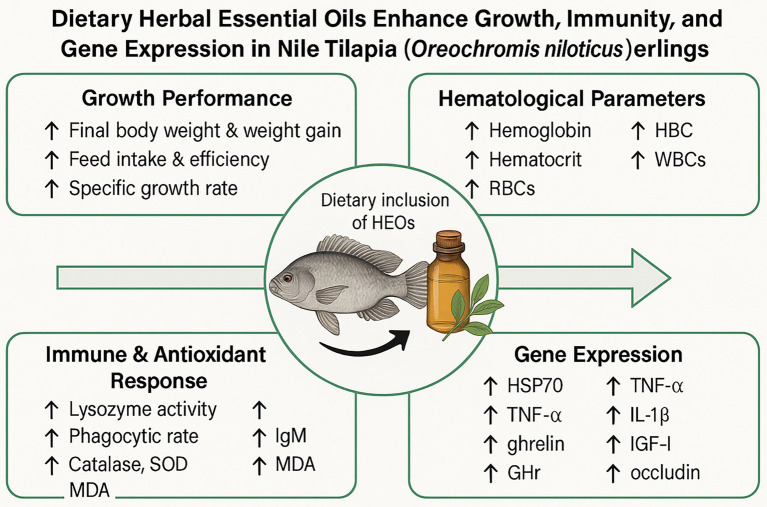

## 1 Introduction

Aquaculture is a rapidly expanding endeavor throughout all continents, rising at an approximate pace of 7% annually, and constitutes over half of the fish consumed by humans (1). Aquaculture farms need to optimize feed ingredients to improve the digestibility of the immune system, decrease FCR microbiota, and reduce fish production costs (2). Freshwater fish farming accounts for over two-thirds of global aquaculture output (3). Nile tilapia is the second most cultivated fish globally, following carp, and accounted for 8.3% of global fish production in 2018 (4).

Sufficient nutrition is essential for sustaining healthy, disease-free fish in diverse aquaculture settings. Natural immunostimulants, growth enhancers, and medicinal herbs are now considered safe and effective alternatives to chemotherapy and antibiotics in aquaculture settings (5). Essential oils (EOs) extracted from plants have been utilized in aquaculture research because they enhance animal health, growth, and welfare (6). Furthermore, incorporating plant-based foods into fish feed is highly intriguing in aquaculture; hence, plant-based extracts should be considered to enhance growth performance and health (probiotics) (7). It has been observed that phenolic chemicals found in essential oils (EOs), including thymol, carvacrol, p-cymene, and ɞ-terpinene, can inhibit both gram-negative and positive pathogens (8). Essential oils include antimicrobial, antiviral, and antifungal characteristics; as a result, they gained popularity as natural feed additives (9). Research on feed additives for farmed fish, including phytogenics, essential oils, prebiotics, and probiotics, has been ongoing for quite some time, and the results have been promising in terms of safety, cost-effectiveness, and environmental friendliness (10).

Essential oils’ many beneficial effects, including their ability to fight cancer, alleviate pain, promote growth, and protect the liver, have increased their use in recent years (11). Immunostimulants derived from medicinal plants, such as spices, seaweeds, and herbs, have a long history of use. They are eco-friendly, cheap, easy to make, and deliver an immune-system-safe substitute for antibiotics and immunoprophylaxis. The active chemicals in these plants possess antibacterial, growth-promoting, immune-enhancing, and stress-relieving characteristics in fish (12, 13).

Better digestion is possible with the help of essential oils since they increase the absorption of broken-down molecules and stimulate enzymes. Antioxidant and antibacterial properties are demonstrated by Hendam et al. (14). Due to their benefits for digestion, gut microbial ecology, growth, and welfare, EOs may 1 day supplement animal diets instead of antibiotics (15). Eos and other natural dietary supplements with bioactive compounds can boost fish’s feed efficiency and growth performance by enhancing digestive secretion (16, 17). Juvenile tilapia fed a diet with OAB had better intestinal health and a higher survival rate (18). Considering that the purpose of this study was to examine the impact on immune response, feed utilization, growth performance, biochemical and hematological variables, and genes related to immunity by supplementing the diet of fingerling Nile tilapia, *O. niloticus*, with a mixture of herbal essential oils (HEOs).

## 2 Materials and methods

### 2.1 Experimental design and fish diets

Five experimental diets were created to meet the specific dietary needs of fingerlings of Nile tilapia, all of which were isonitrogenous and had the same amount of calories. Five groups were assigned different diets: Group 1 (G1, HEOs 0 mL/kg) received no chemicals, and Groups 2 (G2, HEOs 30 mL/kg) and 3 (G3, HEOs 60 mL/kg) received 30 and 60 mL of herbal essential oils (HEOs) per kilogram of feed, respectively. Supplementation levels were increased for Groups 4 (G4, HEOs 120 mL/kg) and 5 (G5, HEOs 240 mL/kg) to 120- and 240-ml HEOs/kg of diet, respectively—Supplementary Table S1 - GC–MS Composition of HEO Blend.

The experimental diets were formulated to be isonitrogenous and isolipidic, with a target of 32% crude protein and 7% lipid. All ingredients were dried to constant weight and mixed on a dry matter (DM) basis. Proximate composition was determined according to AoOA Chemists and AoOA Chemists (19) methods: crude protein by the Kjeldahl method (Method 2001.11), crude lipid by ether extraction (Method 920.39), ash by incineration at 550°C (Method 942.05), and moisture by oven drying at 105°C (Method 930.15).

The basic elements of the diet include fish meal (the main source of animal protein), wheat bran, rice bran, wheat flour, soybean meal, corn gluten, yellow maize, di-calcium phosphate, fish oil, limestone, and a premixed vitamin and mineral blend. Five equal quantities of the materials were measured for each treatment after thorough mixing Table 1.

**Table 1:** Diet formulation and Chemical composition of the basal test diets.

Ingredients (%)	(%)	Chemical composition	(%)
Fish meal (63% crude protein)	10	Dry matter	90.72
Soybean meal (46% CP)	40	Total protein	30.14
Wheat bran	10	Crude lipid	6.5
Rice bran	10	fiber	4.80
Wheat flour	6.9	ash	7.2
yellow corn	13	NFE	51.36
Corn gluten meal (60% CP)	4	Gross energy (Kcal/100 g)2	442.80
Fish oil	3	Digestible energy (kcal/100 g)	390
Vitamin and mineral mix^1^	2	Lysine	1.41
Dicalcium phosphate	1	Methionine	0.58
Limestone	0.1	Threonine	1.05
Total	100	Arginine	1.69
		Isoleucine	1.18
		Leucine	2.03
		Valine	1.35
		Histidine	0.82
		Phenylalanine	1.46
		Tryptophan	0.27

^1^Vitamin mixture (mg/kg premix, free of vitamin C): vitamin A (3300 IU), vitamin D3 (410 IU), vitamin E (2660 mg), vitamin B1 (133 mg), vitamin B2 (580 mg), vitamin B6 (410 mg), vitamin B12 (50 mg), biotin (150 mg), Colin chloride (4000 mg), inositol (330 mg), Para- amino benzoic acid (9330 mg), niacin (26.60 mg), pantothenic acid (2000 mg). Mineral mixture (mg/kg premix): manganese (325 mg), iron (200 mg), copper (25 mg), iodine (10 mg), cobalt (5 mg). ^2^*Gross energy was calculated according to NRC (1993) by using factors of 5.65, 9.45, and 4.22 Kcal per gram of protein, EE, and NFE, respectively.

The liquid herbal essential oils (TRI VIR, Tri Pharma Company, Egypt) were added to each treatment diet in a specific dosage. These oils included carvacrol (45 g/L), oregano oil (45 g/L), 1,8-cineole (16 g/L), thymol (39.2 g/L), *α*-pinene (4.6 g/L), *β*-pinene (2.6 g/L), limonene (3 g/L), and propylene glycol (150 g/L). Each batch was mixed with fish oil after the HEOs were added, and then 400 cc of water per kilogram of feed was added to make a homogenous dough. To make pellets that are just right for fingerlings, this dough was run through laboratory pelleting equipment fitted with a 2 mm die. The pellets were left to dry at room temperature for a full day before being sealed in plastic bags and kept at 4°C until needed. We used industry-standard methods to examine the diets’ chemical makeup (20).

### 2.2 Experimental setup

Nile tilapia were collected from a freshwater farm with no prior health issues or mortality. Fish were sedated onsite using MS-222 (40 mg/L, Syndel, Canada), then transported in labeled plastic bags containing one-third clean water and two-thirds pure oxygen. Upon arrival at the Animal Health Research Institute, Agriculture Research Center, Kafrelsheikh, Egypt, fish underwent a 10-min iodine bath (20 ppm Betadine®, 5% povidone-iodine, Nile Company for Pharmaceuticals) (21).

At first, the Nile tilapia fingerlings were kept in a 1,000-liter fiberglass tank at the Sakha Aquaculture Research unit after being acquired from a private fish farm in Kafrelsheikh, Egypt. Fifteen days before the trial’s start, the fish were given a commercial meal with 30 % crude protein to help them adjust to the lab environment. After acclimatization, 300 fingerlings (27.20 ± 0.06 g) were accidentally allocated to 30 glass aquaria (each with a capacity of 60 liters), with 10 fish stocked per tank. Three aquaria were designated for each dietary treatment, and all tanks were continuously aerated using electric air pumps. The photoperiod was maintained at 12 h light and 12 h dark using programmable LED lighting. Initial biomass density was approximately 4.5 kg/m^3^ (10 fish averaging 27.2 g per 60-L tank). Dissolved oxygen was measured daily at 9:00 a.m. and 5:00 p.m. using a DO meter (YSI Professional Plus), and ranged from 6.5 to 7.0 mg/L across all tanks. Tank positions were randomized at the beginning of the experiment using a computer-generated random number sequence and re-randomized weekly to eliminate potential positional or environmental bias (light, airflow). Dechlorinated water was used throughout the study. Waste was removed daily by siphoning, with 50% of the water in each tank replaced daily, and a full water change was carried out once weekly following cleaning. Fish were fed the experimental diets twice daily—at 8:00 a.m. and 2:00 p.m.—for 72 days. Feeding was based on 3% of the fish’s body weight and was adjusted biweekly according to growth. Water quality parameters were regularly monitored, with the following average values recorded: temperature at 28.53 ± 0.01°C, dissolved oxygen at 6.73 ± 0.14 mg/L, pH at 7.62 ± 0.1, and total ammonia nitrogen at 0.38 ± 0.02 mg/L.

### 2.3 Growth and feed efficiency

The duration of the feeding trial was 72 days. Each fish in each tank was weighed before and after the experiment to see how much they had grown. The fish were fasted for 24 h before the final sampling to reduce stress. The following equations were used to assess the efficiency of feed use and growth performance:

1. Final Weight Gain (%) = ((Final Mean Body Weight–Initial Mean Body Weight)/Initial Mean Body Weight) × 100
2. Weight Gain (%) = 100 × (Final Average Body Weight− Initial Average Body Weight)/Initial Average Body Weight
3. Daily Weight Gain (g/fish/day) = Total Weight Gain(g)/Duration of Experiment (days)
4. Specific Growth Rate (SGR; %/day) = {ln (Final Body Weight) − ln (Initial Body Weight)} × 100/Number of Days, where ln is the natural logarithm.
5. Feed Conversion Ratio (FCR) = Dry Feed Intake(g)/Wet Weight Gain(g)
6. Protein Efficiency Ratio (PER) = Weigh Gain(g)/Protein Intake(g)
7. Survival Rate (%) = (Final Number of Fish/Initial Number of Fish) × 100

### 2.4 Sampling

Prior to sampling, fish were fasted for 24 h to eliminate postprandial effects on hematological and biochemical parameters. Blood was collected from the caudal vein using 1 mL heparinized syringes under anesthetic conditions.

Fish blood was drawn from the caudal vertebral vein at the end of the experiment using sterilized hypodermic syringes and an anesthetic. Blood samples were separated into two tubes. Hematological tests were performed in the first tube using EDTA as an anticoagulant. The second tube without an anticoagulant was left at room temperature for 3 h to allow clot formation. After centrifuging the clotted blood at 3000 RPM for 15 min, serum was extracted. The serum was stored at −20°C until biochemical analysis.

#### 2.4.1 Determination of haemato-immunological variables

Hb, WBC, and (RBC) counts were all measured following an established protocol (22). The differential leukocytic counts were performed using Giemsa-stained blood smears. The microhematocrit technique is used to determine the packed cell volume (PCV).

#### 2.4.2 Determination of blood biochemical variables, antioxidative status, and immunity

Serum total protein and albumin concentrations were measured using commercially available colorimetric assay kits, following the manufacturer’s protocols (23, 24).

By subtracting albumin values from total protein measurements, globulin levels were estimated. The RA-50 semi-automated chemistry analyzer (Bayer) was used to measure several biochemical parameters in the blood, such as amylase and lipase enzyme activity (AST and ALT), immunoglobulin M (IgM), creatinine, urea, uric acid, total cholesterol, and triglycerides. The biochemical assays were carried out using diagnostic kits provided by Spinreact Co., Spain, following the manufacturer-specified protocols.

A turbidimetric method was used to assess the activity of serum lysozyme (25), produced by studying the gram-positive bacteria *Micrococcus lysodeikticus* (Sigma, United States). We used the following approaches to evaluate leukocyte phagocytes (26). The phagocytosis assay smear was used to quantify the amount of leukocytes that absorbed bacteria as a ratio to the total leukocyte count. The phagocytic index and phagocytic activity were evaluated according to the methods described by Kawahara et al. (27).

Serum (MDA) concentrations were assessed with the thiobarbituric acid assay. The samples were calorimetrically cleared utilizing a commercial kit (LPO 586 Kit, OXIS International Inc., Portland, United States) as outlined by Ohkawa et al. (28). The serum (SOD) measurement follows McCord and Fridovich (29), and (CAT) following Aebi (30) using (Biodiagnostic Co., Giza, Egypt).

### 2.5 Gene expression

The Quantitative gene expression analysis was done using real-time reverse transcription PCR (RT-PCR). The genes EF1-*α* and *β*-actin served as internal controls for data normalization (primer sequences are provided in Table 2). Total RNA was isolated from tissue samples using Trizol reagent (iNtRON Biotechnology), following the manufacturer-supplied protocol. The integrity of RNA was confirmed through electrophoresis on a 2% agarose gel, and concentrations were determined using a Nanodrop spectrophotometer (Quawell, United States).

**Table 2:** Primers used for qRT-PCR analysis.

Gene symbol	Primer sequence		Accession number	Efficiency (%)	*R*^2^ value
Ghrelin	F	GTGGTGCAAGTCAACCAGTG	MW556311.1	100.1	0.996
	R	CATGGCTTGGCGACCAATTC			
SOD	F	CTCCAGCCTGCCCTCAA	JF801727.1	94.9	0.997
	R	TCCAGAAGATGGTGTGGTTAATGTG			
*TNF-α*	F	GGAAGCAGCTCCACTCTGATGA	JF957373.1	101.8	0.996
	R	CACAGCGTGTCTCCTTCGTTCA			
CAT	F	TCCTGGAGCCTCAGCCAT	JF801726.1	96.8	0.997
	R	ACAGTTATCACACAGGTGCATCTTT			
*IL-1β*	F	CAAGGATGACGACAAGCCAACC	XM_003460625.2	98.8	0.998
	R	AGCGGACAGACATGAGAGTGC			
Occludin	F	AATCGGGATAATCTCCTACA	XM_003445131.5	100.9	0.999
	R	TTGGTCCTCTTTGCTATTTG			
*zo-1*	F	CCGCAGATCAGTCCCTCTTC	XM_013270540.3	97.6	0.999
	R	GTACGGAGTTAGCATCGCCA			
hsp70	F	CATCGCCTACGGTCTGGACAA	FJ207463.1	98.4	0.996
	R	TGCCGTCTTCAATGGTCAGGAT			
IGF-1	F	GCAGATTGCTGATGGCATGG	KC506777	99.7	0.997
	R	TCATTCCGAAGTCGCCGAT			
GHr1	F	TCCGCTGCAGATGGAATGTT	NM_001279455	96.1	0.999
	R	AAAAGCACTCGTTTGGCGTC			
β-actin	F	TGACCTCACAGACTACCTCATG	XM_003443127.5	99.3	0.997
	R	TGATGTCACGCACGATTTCC			
EF1-α		TGATCTACAAGTGCGGAGGAA	AB075952.1	100.5	0.995
		GGAGCCCTTTCCCATCTCA			

To synthesize cDNA, 2 μg of high-quality total RNA from each sample was reverse transcribed using a Bioline cDNA synthesis kit (United Kingdom), adhering to the manufacturer’s recommendations. PCR amplification was conducted using the SensiFast SYBR Lo-Rox kit (Bioline) in a 20 μL total volume. Each reaction included 2 μL of cDNA template, 10 μL of SYBR Green master mix, and 0.5 μM of each primer.

The thermal cycling program included an initial denaturation at 95°C for 10 min, followed by 40 cycles of denaturation at 95°C for 15 s and annealing/extension at 60°C for 1 min. Amplification data and Ct values were collected automatically using the Rotor-Gene Q system (Qiagen, Valencia, CA, United States). The relative quantification of target gene expression was calculated using the 2^−ΔΔCt method (31). To validate the stability of the two reference genes (EF1-*α* and *β*-actin), expression data were analyzed using **geNorm** and **NormFinder** software tools. The geNorm analysis revealed M-values of 0.41 (EF1-α) and 0.44 (β-actin), both below the accepted threshold of 1.5, indicating stable expression under our experimental conditions. NormFinder also ranked these genes as the two most stable among the four candidates tested (including GAPDH and 18S rRNA; data not shown), with a combined stability value = 0.28. Primer efficiency was calculated from standard curves generated by serial 10-fold dilutions of pooled cDNA. All primer pairs exhibited efficiencies between **95 and 104%** and *R*^2^ values > 0.99.

### 2.6 Histological and morphometric analysis of intestinal villi

Five randomly selected fish from each treatment (HEOs 0, 30, 60, 120, and 240 mL/kg) were histopathologically examined following deep anesthesia with 40% ethyl alcohol. Tissue samples were taken from the front, middle, and posterior intestines. The materials were dehydrated in 70–100% ethyl alcohol after a one- to two-day fixation in 10% formaldehyde. After dehydration, samples were paraffin waxed and xylene washed. According to histopathological and morphometric analysis techniques, Hematoxylin and eosin staining were performed on 4–5 μm sections (32, 33). Image analysis software (NIH, Bethesda, MD) evaluated intestinal villi length, breadth, and crypt depth. Each set of five intestinal cross-sections had 10 randomly selected villi and crypts, which were examined using one-way ANOVA (SPSS version 22, SPSS Inc., IL, United States). A *p*-value under 0.05 was significant. Although five fish per treatment were histologically examined, we confirm that three non-overlapping intestinal sections per fish were evaluated to increase statistical robustness. This results in 15 observations per treatment group for morphometric analysis.

### 2.7 Statistical model

All statistical analyses were conducted using SPSS version 22 (IBM, United States). The experimental unit for all analyses was the tank (*n* = 3 per treatment), and tank-level means were used to avoid pseudoreplication.

Data were first checked for normality using the Shapiro–Wilk test and for homogeneity of variances using Levene’s test. When assumptions were met, one-way analysis of variance (ANOVA) was used to detect differences among treatments, followed by means were compared using the Tukey–Kramer *post hoc* test to account for unequal sample sizes. The level of significance was set at *α* = 0.05.

In addition, a linear mixed model (LMM) was fitted using the `PROC MIXED` procedure to account for random tank effects. The model equation was:


Y_ij=μ+T_i+e_ij

Where Y_ij is the observed response in tank j of treatment i, μ is the overall mean, T_i is the fixed effect of treatment, and e_ij is the residual error (random). A variance components (VC) covariance structure was used. Model fit was assessed using Akaike Information Criterion (AIC) and residual plots. Residuals were confirmed to be normally distributed with homoscedasticity. For key response variables (e.g., SGR, FCR, GHr, IGF-I), both one-way ANOVA and LMM approaches yielded consistent results.

### 2.8 Sample size and power consideration

A total of 15 tanks (3 per treatment group) were used in the study. *Post hoc* power analysis was performed using G*Power version 3.1.9.7 to assess whether this design had sufficient sensitivity to detect significant treatment effects. Using a one-way ANOVA model with five groups, *α* = 0.05, and n = 3 replicates per group, the analysis showed that effect sizes of *f* = 0.40–0.50 (based on observed SGR and gene expression differences) yielded power levels >0.80. This indicates the sample size was sufficient for detecting large and biologically relevant treatment effects.

## 3 Results

### 3.1 Efficiency in growth, feed consumption, and mortality rate

At the end of the 72-day feeding experiment, significant improvements in growth performance metrics were noted in Nile tilapia (*p* ≤ 0.05), as outlined in Table 3. The initial body weight (IBW) of fish across all treatment groups showed no statistical differences (*p* =0.654), indicating that all groups began the trial under equivalent conditions.

**Table 3:** Effect of HEOs combination (ml/kg) on the growth performance of fingerlings Nile tilapia (*O. niloticus*).

Item	G1 0 ml/kg	G2 30 mL/kg	G3 60 ml/kg	G4 120 ml/kg	G5 240 ml/kg	Pooled SEM
IBW (g)	27.03	27.3	27.26	27.1	27.33	0.15
FBW (g)	54.06^bc^	59.83^ab^	64.99^a^	51.88^c^	50.08^c^	2.08
WG (%)	99.97^bc^	119.24^ab^	138.54^a^	91.60^bc^	83.21^c^	2.73
DWG	0.37^bc^	0.45^ab^	0.52^a^	0.34^c^	0.31^c^	0.02
SGR (%/day)	0.96^b^	1.08^ab^	1.20^a^	0.90^c^	0.84^c^	0.05
FI (g/fish/72 days)	66.11^bc^	70.82^ab^	74.25^a^	62.55^c^	59.65^c^	2.69
FCR	2.45^ab^	2.18^b^	1.98^c^	2.54^a^	2.63^a^	0.1
FE	0.40^bc^	0.45^ab^	0.50^a^	0.39^c^	0.38^c^	0.01
Survival (%)	100.0^a^	100.0^a^	93.33^b^	90.00^b^	80.00^c^	1.49
PER	1.35^bc^	1.53^ab^	1.67^a^	1.29^c^	1.26^d^	0.06
PPV	18.11^ab^	21.23^a^	22.72^a^	18.20^ab^	16.81^c^	1.51

Data are presented as mean ± SE of three replicate tanks (*n* = 3 per treatment). Statistical analysis was performed using one-way ANOVA on tank-level means. In the same row, different letters differ significantly (*p* ≤ 0.05). IBW, initial body weight; FBW, final body weight; WG, weight gain; SGR, specific growth rate; FI, feed intake; FCR, feed conversion ratio.

Tilapia-fed diets enriched with 30 or 60 mL/kg of herbal essential oils (HEOs) achieved the best growth responses, recording significantly higher values for final body weight (FBW), weight gain percentage (WG%), daily weight gain (DWG), feed intake (FI), feed efficiency (FE), and specific growth rate (SGR) compared to the other groups (*p* =0.0241).

On the other hand, the poorest growth outcomes were observed in the control group, and fish fed higher HEO inclusion levels (120 and 240 mL/kg). Feed conversion ratio (FCR) was significantly improved (i.e., reduced) in the 30 and 60 mL/kg HEO groups, whereas the control and high-inclusion groups did not show significant changes in FCR. Furthermore, both protein efficiency ratio (PER) and protein productive value (PPV) were substantially higher in the 30 and 60 mL/kg treatment groups than in the control or higher-dose groups (*p* =0.0145). Survival rates were also significantly better in the control group and those fed 30 and 60 mL/kg of HEOs, showing a noticeable decline in the groups receiving 120 and 240 mL/kg (*p* =0.0214).

### 3.2 Haemato-biochemical parameters

Introducing HEOs substantially impacted blood hematological and biochemical parameters (*p* =0.0324). Table 4 shows that, associated with the control group, the Nile tilapia fish given a diet with 30, 60, 120, and 240 mL HEOs/kg−1 diet had increased levels of hemoglobin, platelet-count value (*p* =0.014), (RBCs), and (WBCs). Nevertheless, the current study did not find any significant impacts of utilizing a combination of HEOs on the blood biochemical features of Nile tilapia (*p* =0.0214; Table 5). Treatment groups also had reduced levels of triglycerides and cholesterol. As a result, they consumed less HEO in their diet compared to the control group and other groups, using a combination of 30 and 60 mL/kg−1 HEO (Table 5).

**Table 4:** Effect of HEOs combination (ml/kg) on the hematological traits of fingerlings Nile tilapia (*O. niloticus*).

Item	G1 0 ml/kg	G2 30 ml/kg	G3 60 ml/kg	G4 120 ml/kg	G5 240 ml/kg	Pooled SEM
Hb (g/100 mL)	13.45 ^a^	14.61 ^a^	14.17 ^a^	11.05 ^b^	13.76 ^a^	0.25
RBCs (10/m m^3^)	4.46 ^a^	4.86 ^a^	4.74 ^a^	3.61 ^b^	4.54 ^a^	0.25
PCV (%)	43.66 ^a^	46.66 ^a^	46.00 ^a^	34.33 ^b^	44.66 ^a^	2.68
MCV (μm^3^/cell)	97.69 ^ab^	95.88 ^b^	97.05 ^ab^	94.59 ^b^	98.38 ^a^	1.05
MCH (pg/cell)	30.12	30.02	29.95	30.54	30.29	0.23
MCHC (%)	30.83	31.32	30.86	32.30	30.79	0.35
WBCs (10/mm^6^)	12.14	11.12	11.36	14.87	15.23	1.45
Heterophils (%)	14.66	10.00	12.00	13.33	18.66	2.7
Lymphocytes (%)	75.66	80.33	78.66	76.33	70.66	2.6
Monocytes (%)	8.00	8.00	7.66	8.67	7.00	0.6
Eosinophils (%)	1.00	1.00	1.00	1.00	1.33	0.3
Basophil (%)	0.66	0.66	1.00	0.66	1.00±	0.36

Data are presented as mean ± SE of three replicate tanks (*n* = 3 per treatment). Statistical analysis was performed using one-way ANOVA on tank-level means. In the same row with different letters, they differ significantly (*p* ≤ 0.05). Hb, hemoglobin; RBCs, red blood cells; PCV, packed cell volume; MCV, mean corpuscular volume; MCH, mean corpuscular hemoglobin; MCHC, mean corpuscular hemoglobin concentration; WBCs, white blood cells.

**Table 5:** Effect of HEOs combination (ml/kg) on blood biochemical traits of fingerlings Nile tilapia (*O. niloticus*).

Item	G1 0 ml/kg	G2 30 ml/kg	G3 60 ml/kg	G4 120 ml/kg	G5 240 ml/kg	Pooled SEM
Glucose (mg/dl)	11.98	12.65	10.48	12.53	13.4	1.28
ALT (U/I)	30.56	28.23	27.50	29.71	31.24	1.55
AST (U/I)	19.73	19.78	18.61	20.89	20.71	1.44
Total protein (g/dl)	4.07	4.41±0.19	4.84±0.10	4.34	3.97	0.27
Albumin (g/dl)	1.39	1.46	1.54	1.43	1.42	0.05
Globulin (g/dl)	2.68	2.94	3.30	2.91	2.55	0.23
Creatinine (mg/dl)	1.04	0.93	0.95	1.00	1.01	0.06
Urea (mg/dl)	1.72	1.35	1.72	1.55	1.40	0.21
T-CHO (mg/dl)	81.32^c^	93.59^b^	92.33^b^	101.45^a^	97.41^a^	2.53
TG (mg/dl)	92.64^b^	90.64^c^	94.98^ab^	97.98^ab^	99.94^a^	1.99

Data are presented as mean ± SE of three replicate tanks (*n* = 3 per treatment). Statistical analysis was performed using one-way ANOVA on tank-level means. In the same row, different letters differ significantly (*p* ≤ 0.05). ALT, alanine aminotransferase; AST, aspartate aminotransferase; T-CHO, total cholesterol; TG, triglycerides.

### 3.3 Immune parameters

There were notable group variations in the fish immune response markers; those groups that consumed higher concentrations of HEO had an improved immune response (Table 6). In fingerlings, lysozyme and phagocytic activity showed a greater value. Unlike the other groups, Nile tilapia *O. niloticus* fed a diet containing 30 and 60 mL HEOs/kg−1 and fared relatively well. Several experimental meals had similar phagocytic index values (*p* =0.756). In the fish-fed diet with HEOs, IgM levels were substantially higher than in the control and fish-fed 60 mL HEOs/kg−1 diet (*p* < 0.05; Table 6).

**Table 6:** Effect of HEOs combination (ml/kg) on Immune and antioxidative responses of fingerlings Nile tilapia *O. niloticus*.

Item	G1 0 ml/kg	G2 30 ml/kg	G3 60 ml/kg	G4 120 ml/kg	G5 240 ml/kg	Pooled SEM
SOD (u/gm)	7.49^ab^	9.05^ab^	10.35^a^	6.39^b^	4.52^c^	0.89
CAT (u/gm)	12.75^ab^	12.19^ab^	16.49^a^	11.59^ab^	8.99^b^	1.77
MDA (nmol/g)	7.54	7.63	7.69	9.20	8.79	0.85
Lysozyme (mg/ml)	4.39^c^	6.36^b^	7.15^a^	4.37^c^	4.03^c^	1.06
Phagocytic activity	8.68^ab^	9.45^a^	9.82^a^	6.63^bc^	5.56^c^	1.82
Phagocytic index	1.09	1.02	1.15	1.05	0.97	0.06
IGM (mg/ml)	4.70^b^	5.42^ab^	7.48^a^	3.53^b^	3.35^b^	0.73

Data are presented as mean ± SE of three replicate tanks (*n* = 3 per treatment). Statistical analysis was performed using one-way ANOVA on tank-level means. In the same row, different letters differ significantly (*p* < 0.05).

### 3.4 Antioxidant enzymes and gene expression in *Oreochromis niloticus*

Feeding Nile tilapia fingerlings with herbal essential oils (HEOs) significantly affected antioxidant enzyme activity (Table 6). Both (SOD) and (CAT) activities were substantially elevated (*p* ≤ 0.05) in fish-fed diets containing HEOs associated with the control group. The MDA levels showed no significant differences across all treatment groups (*p* =0.6547).

**Figure 1:**
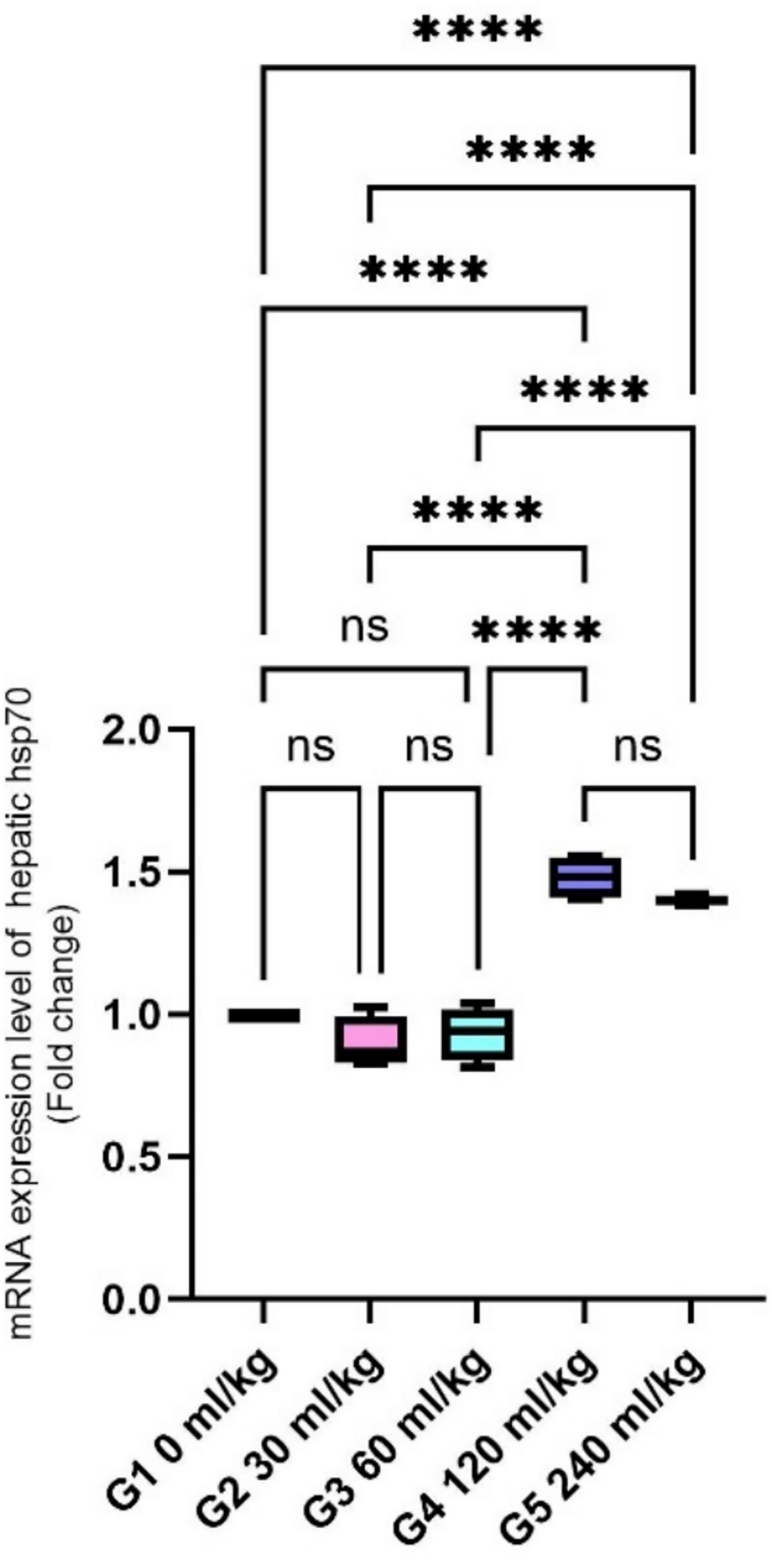
Effect of HEOs supplementation on the expression levels of HSP70 in fingerlings of Nile tilapia *O. niloticus.* HSP70: heat shock protein. Data are mean ± SE; values with different superscripts in the same column differ (*p* < 0.05).

Regarding gene expression, the relative mRNA levels of HSP70 were substantially reduced in fish fed 30 and 60 mL/kg of HEOs (*p* =0.0145). In contrast, fish receiving 120 and 240 mL/kg diets exhibited a significant upregulation of HSP70 expression (Figure 1).

**Figure 2:**
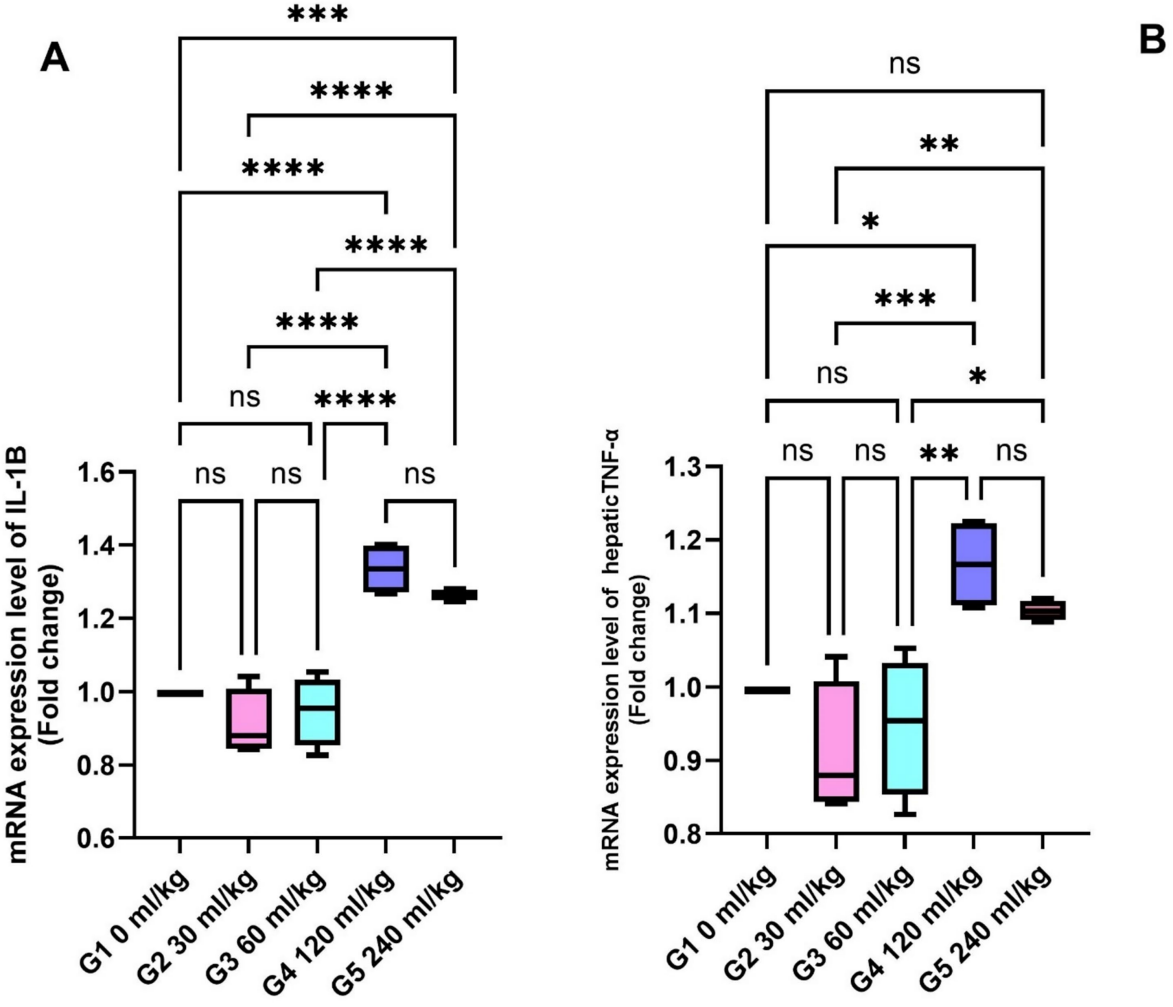
Effect of HEOs supplementation on the expression levels of **(A)** IL-1*β*, and **(B)** TNF-*α* in fingerlings Nile tilapia *O. niloticus.* IL-1β: interleukin1β and tumor necrosis factor alpha (TNF-α). Data are mean ± SE; values with different superscripts in the same column differ (*p* < 0.05).

Fish fed 30 and 60 mL/kg HEOs showed lower IL-1β and TNF-*α* levels than other dietary groups (*p* =0.014; Figures 2A,B), indicating reduced inflammatory activity.

**Figure 3:**
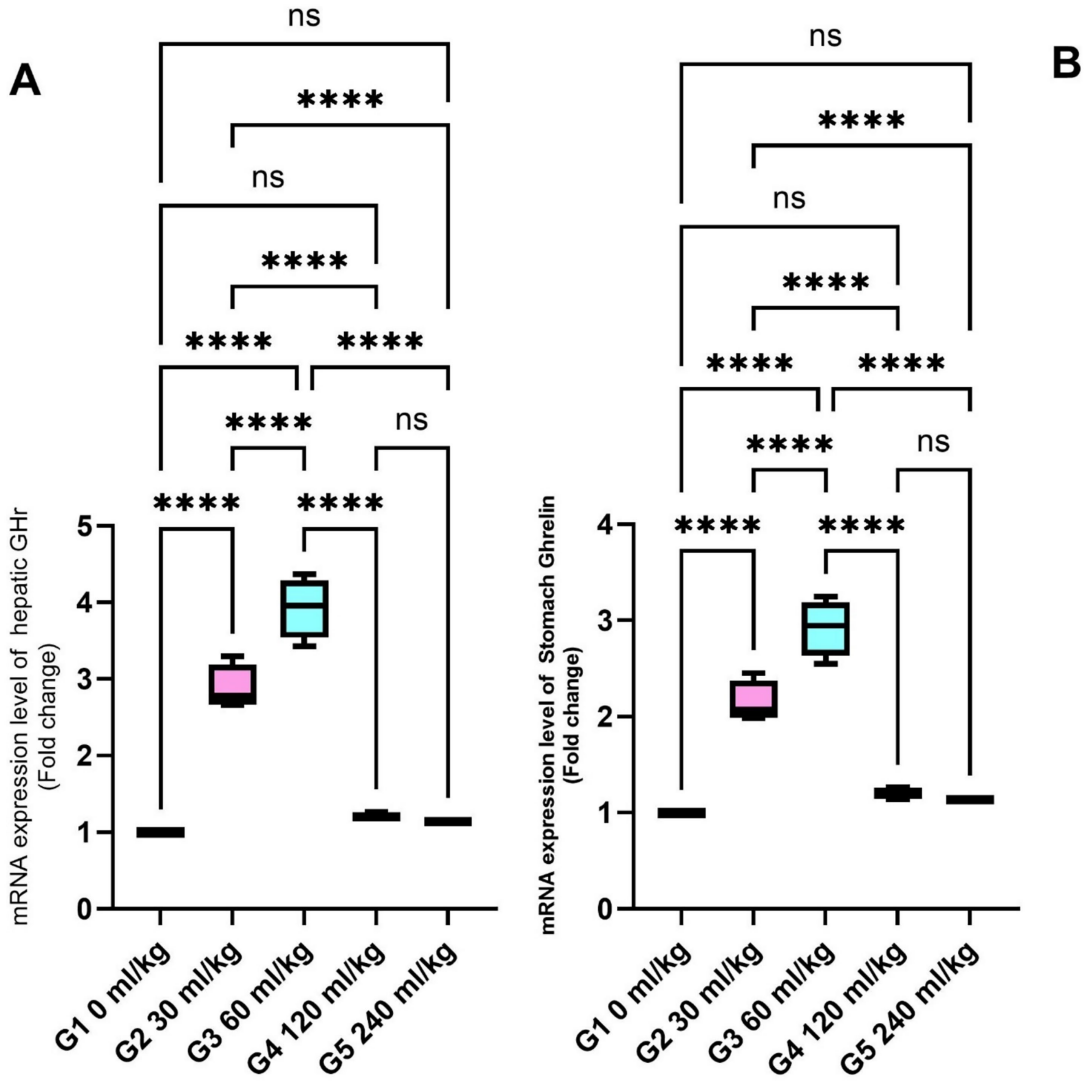
Effect of HEOs supplementation on the expression levels of **(A)** GH and **(B)** Ghrelin in fingerlings Nile tilapia *O. niloticus.* GH is a growth hormone, and Ghrelin is a peptide hormone. Data are mean ± SE; values with different superscripts in the same column differ (*p* < 0.05).

The expression of (GHr) in liver tissue was significantly upregulated in fish supplemented with 30 and 60 mL/kg of HEOs (Figure 3A), while ghrelin—a hormone associated with metabolism and appetite regulation—was also significantly more expressed in the stomachs of fish from these groups (*p* =0.017; Figure 3B).

**Figure 4:**
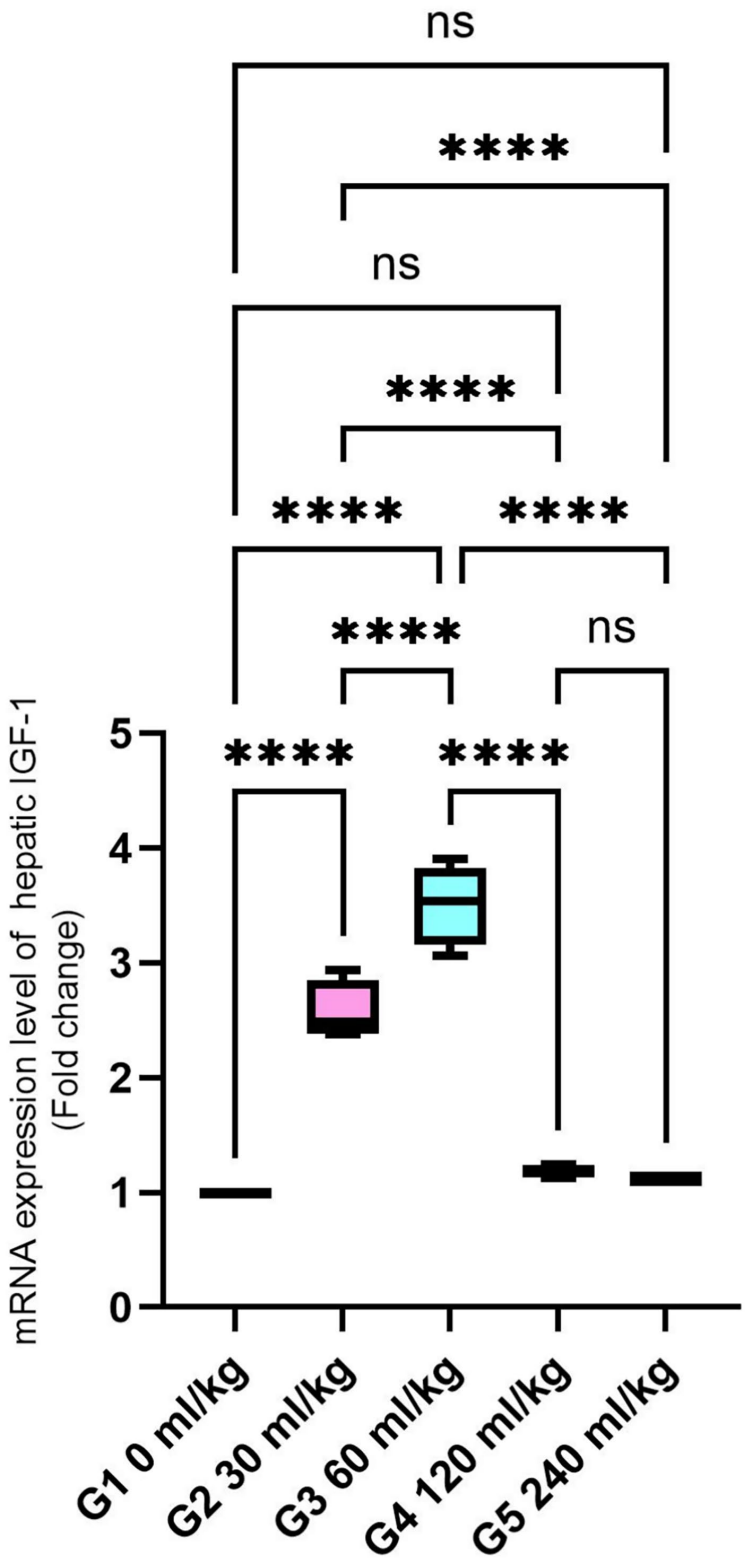
Effect of HEOs supplementation on the expression levels of IGF-I in fingerlings of Nile tilapia *O. niloticus.* Insulin-like growth factor 1 (IGF-I) Data are mean ± SE; values with different superscripts in the same column are different (p < 0.05).

Additionally, *IGF-I* gene expression in the liver was significantly higher in fish-fed diets containing 30 and 60 mL/kg of HEOs than in the other groups (*p* =0.025; Figure 4), suggesting enhanced growth signaling.

**Figure 5:**
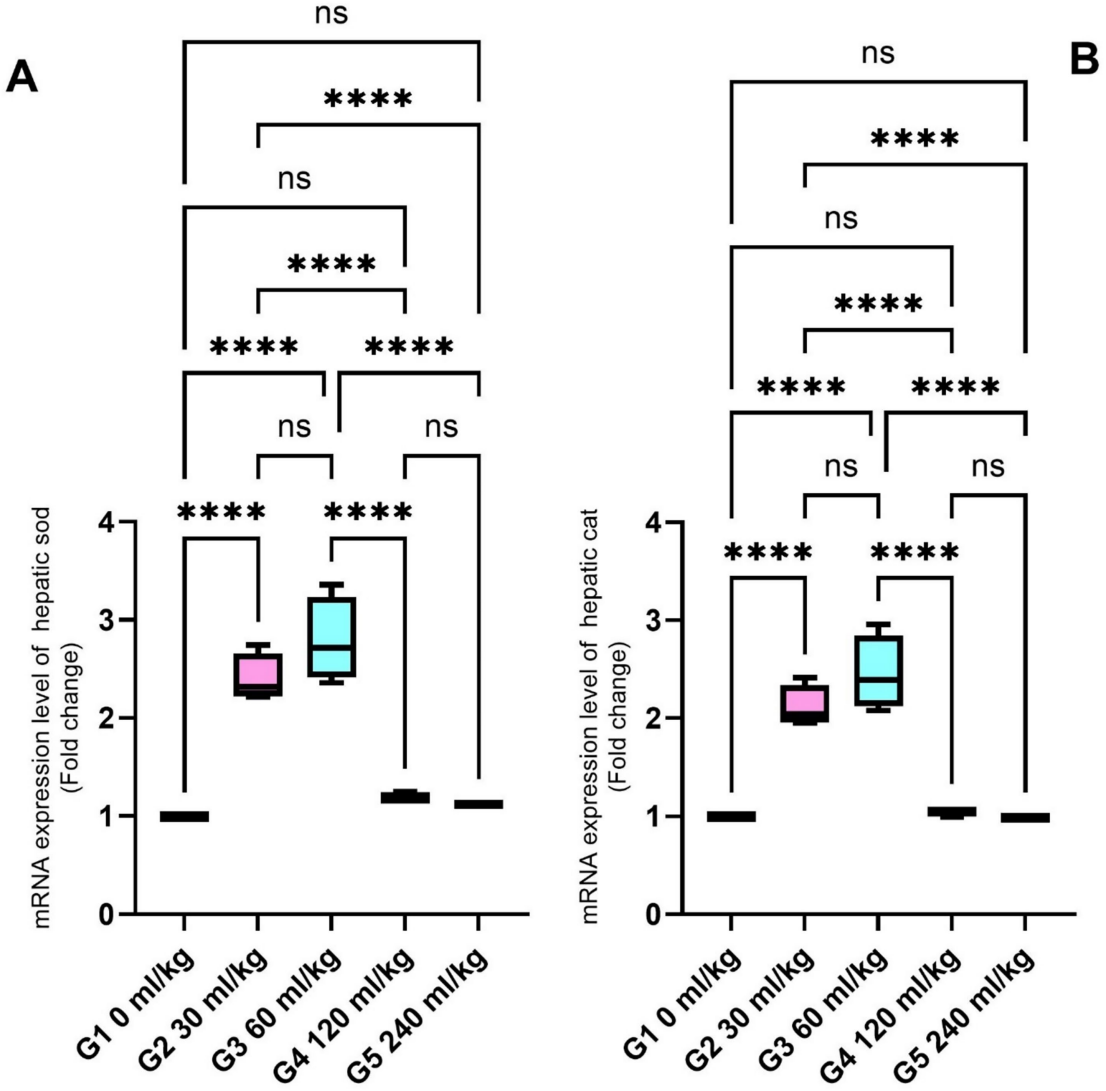
Effect of HEOs supplementation on the expression levels of **(A)** SOD and **(B)** CAT in fingerlings of Nile tilapia *O. niloticus.* Superoxide dismutase (SOD) and catalase (CAT). Data are mean ± SE; values with different superscripts in the same column differ (*p* < 0.05).

**Figure 6:**
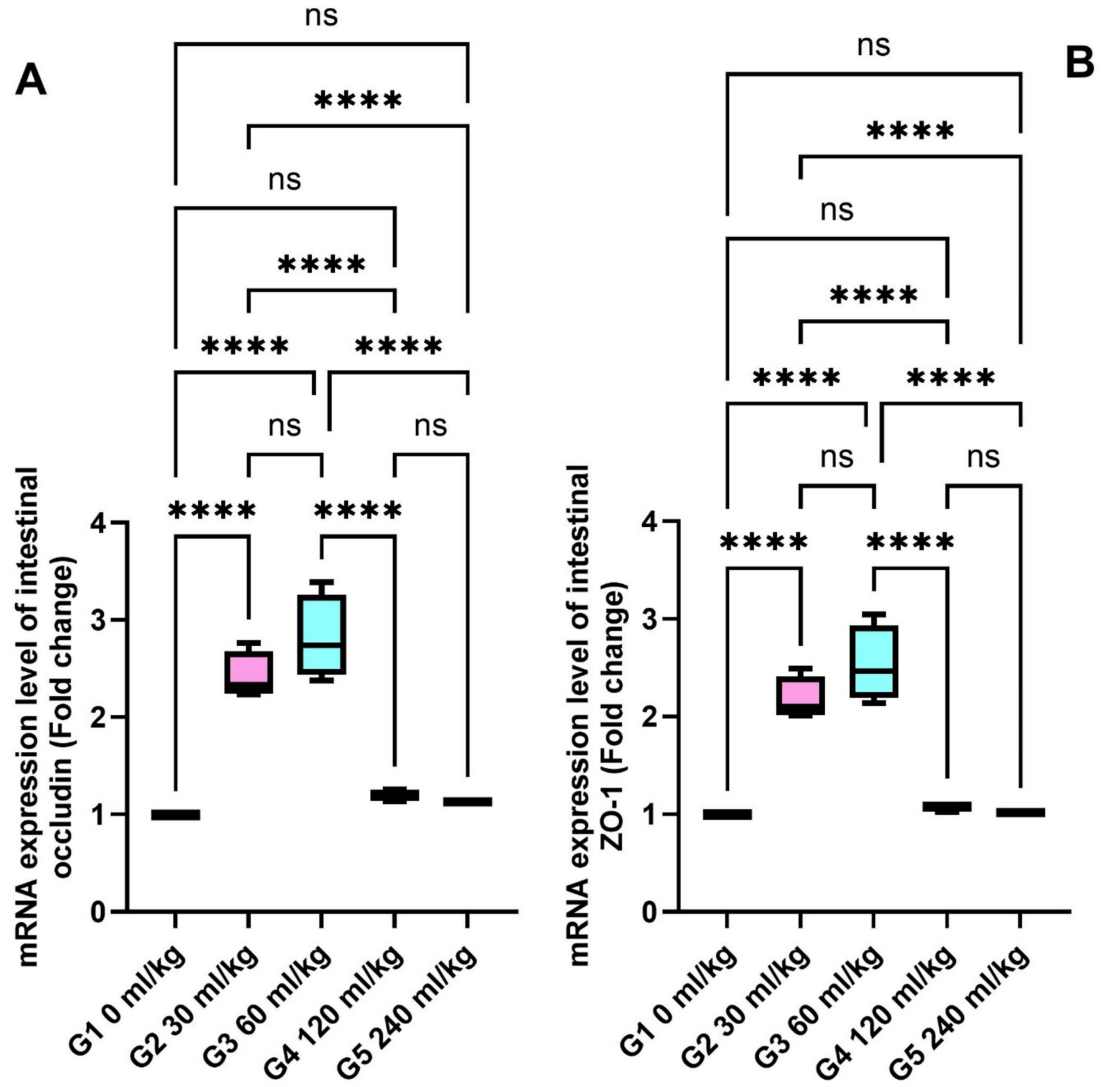
Effect of HEOs supplementation on the expression levels of **(A)** ZO-1 and **(B)** occludin in fingerlings Nile tilapia *O.niloticus.* Zonula occludens-1 (ZO-1) and occludin. Data are mean ± SE; values with different superscripts in the same column differ (*p* < 0.05).

SOD and CAT were also significantly increased in the liver tissues of fish fed 30 and 60 mL/kg of HEOs (*p* ≤ 0.05; Figures 5A,B), consistent with the enzyme activity results. Zonula occludin-1 (ZO-1) and occludin showed significant increases in the groups receiving 30 and 60 mL/kg of HEOs, with the greatest expression detected in the 60 mL/kg group (*p* =0.036; Figures 6A,B), indicating improved gut barrier integrity.

### 3.5 Histopathological findings

**Figure 7:**
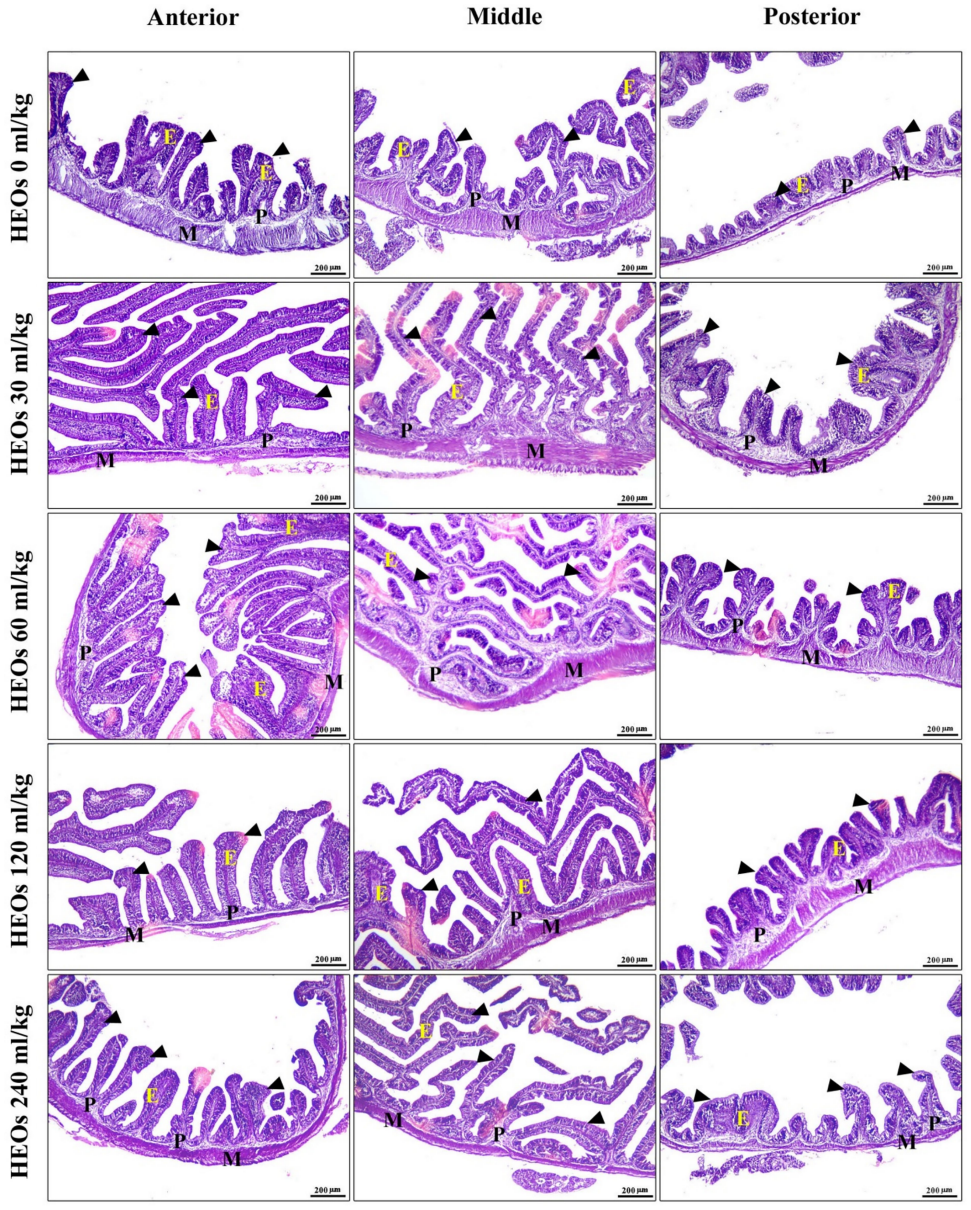
photomicrograph of H&E-stained panel of anterior, middle, and posterior parts of intestine of HEOs 0, 30, 60, 120, and 240 mL/kg groups showing intestinal villi (arrowheads) lined by simple columnar epithelium of lamina epithelialis (E), lamina propria (P), and lamina muscularis (M).

Many intestinal villi are bordered by simple columnar epithelium containing goblet cells, and the intestinal wall seems intact in all three types. Their lamina muscularis and tunica serosa are external coverings for a core of loose connective tissue. Compared to other groups, the intestinal villi in the 30 mL/kg and 60 mL/kg HEOs groups exhibit high branching. Villi length, surface area, and villi length/crypt depth were all significantly increased in the 30 mL/kg and 60 mL/kg HEO groups compared to the 0 mL/kg, 120 mL/kg, and 240 mL/kg groups in the morphometric analysis of data acquired from the measurement of intestinal villi. In Table 7, you can see a summary of the results of the morphological analysis in Figure 7 and Supplementary Table S2.

**Table 7:** Morphometric analysis of the intestine of *O. niloticus* of different groups.

Groups	G1 0 ml/kg	G2 30 ml/kg	G3 60 ml/kg	G4 120 ml/kg	G5 240 ml/kg	*p* value	Pooled SEM
Parameters							
Anterior part							
Villi length (μm)	431.8^b^	589.8^a^	595.5^a^	495.1^ab^	459.4^b^	0.0014	25.75
Crypt depth (μm)	63.68	57.63	74.09	59.27	53.63	0.0764	4.96
villi width (μm)	103.7	133.1	115.0	135.4	106.2	0.0171	7.54
Villi length/crypt depth	6.99^b^	9.69^a^	9.87^a^	8.09^ab^	8.05^ab^	0.0111	0.58
Villi surface area (μm^2^)	54,744^b^	90,453^a^	84,565^a^	73,335^ab^	52,085^b^	0.0042	683.45
Middle part							
Villi length (μm)	360.5^c^	842.5^a^	758.2^a^	543.2^b^	618.2^b^	<0.0001	30.93
Crypt depth (μm)	69.98	88.12	108.6	80.19	71.89	0.0572	9.46
villi width (μm)	144.4	145.9	121.8	121.4	122.0	0.0083	5.79
Villi length/crypt depth	5.95^b^	9.71^a^	9.58^a^	7.81^ab^	8.48^ab^	0.0033	0.65
Villi surface area (μm^2^)	50,979^c^	109,938^a^	111,147^a^	64,901^bc^	81,193^b^	<0.0001	683.18
Posterior part							
Villi length (μm)	172.9^c^	299.0^b^	356.3^a^	243.8^b^	282.5^b^	<0.0001	13.22
Crypt depth (μm)	51.76	70.83	63.18	74.58	62.10	0.0694	5.51
villi width (μm)	118.9	143.4	131.5	108.3	140.7	0.0492	8.74
Villi length/crypt depth	3.44^c^	5.02^ab^	5.69^a^	4.12^bc^	4.69^abc^	0.0027	0.36
Villi surface area (μm^2^)	24,624^c^	47,245^ab^	64,753^a^	37,413^bc^	47,609^ab^	<0.0001	690.77

Data are presented as mean ± SE of three replicate tanks (*n* = 3 per treatment). Statistical analysis was performed using one-way ANOVA on tank-level means. The values with different superscripts in the same row indicate significant differences (*p*<0.05).

### 3.6 Dose–response modeling

To determine the optimal inclusion level of HEOs, broken-line and quadratic regression analyses were performed for specific growth rate (SGR), feed conversion ratio (FCR), and expression of GHr and IGF-I. The broken-line model estimated the optimal HEO inclusion level at 52.6 mL/kg diet for SGR and 55.4 mL/kg for FCR. Beyond this threshold, no further performance improvements were observed, and higher levels were associated with reduced gains.

**Figure 8:**
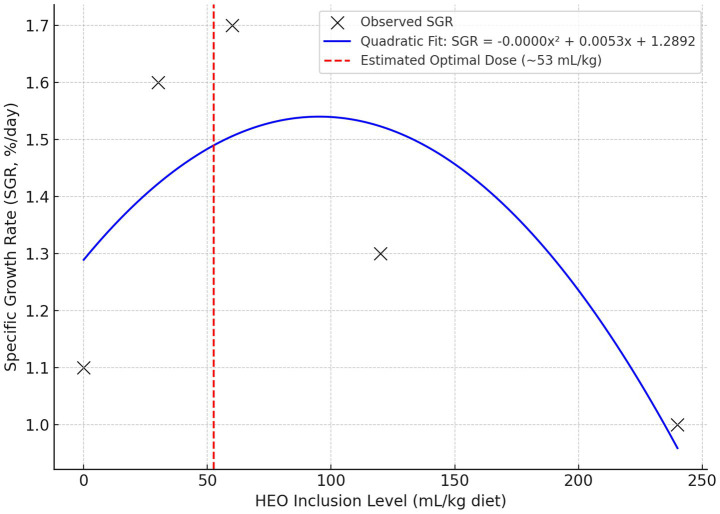
The quadratic relationship between HEO dose and specific growth rate (SGR), estimating the optimal dose near 53 mL/kg.

These results are illustrated in Figure 8 and support our conclusion that the optimal HEO dose lies between 50 and 60 mL/kg.

## 4 Discussion

This study demonstrated that supplementing Nile tilapia diets with 30 and 60 mL/kg of blended herbal essential oils (HEOs) significantly improved growth performance metrics, including final body weight (FBW), specific growth rate (SGR), and feed conversion ratio (FCR). These enhancements can be mechanistically linked to the bioactive components of the HEO blend—particularly carvacrol, thymol, and 1,8-cineole—which are known to stimulate appetite, enhance digestive enzyme secretion, and improve intestinal morphology. Carvacrol and thymol, for example, modulate gut microbiota and reduce pathogenic load, promoting better nutrient absorption and feed utilization. These functional properties may explain the improved protein efficiency ratio (PER) and feed efficiency observed in our study. Additionally, 1,8-cineole has been shown to exert anti-inflammatory effects in the gut, which may reduce energy diversion toward immune responses and favor growth. By enhancing both digestive function and gut integrity, the HEO blend supported more efficient nutrient metabolism and somatic growth, consistent with findings reported in Nile tilapia and other aquaculture species (34, 35).

Supplementation with 30 and 60 mL/kg of HEOs significantly increased hemoglobin (Hb), red blood cells (RBCs), and white blood cells (WBCs), reflecting improved oxygen transport and immunocompetence. These hematological responses are likely driven by the stimulatory effects of thymol and carvacrol on hematopoiesis and leukocyte activation. Additionally, the observed reduction in serum cholesterol and triglycerides may be attributed to the hypolipidemic effects of carvacrol and limonene, which modulate lipid metabolism through inhibition of key hepatic enzymes. Importantly, liver and kidney markers (ALT, AST, urea, and creatinine) remained stable, indicating no adverse systemic effects. These results suggest that HEOs not only enhance blood health but also help maintain metabolic balance under physiological conditions, aligning with findings from El-Bab et al. (9).

Although moderate inclusion levels of HEOs (30–60 mL kg^−1^) significantly improved growth and immune performance, higher doses (≥120 mL kg^−1^) resulted in depressed growth, lower feed intake, and slightly reduced survival. Several factors may explain this dose-dependent response. First, the high concentrations of volatile compounds such as carvacrol and thymol may reduce feed palatability due to strong odor or flavor, leading to reduced voluntary intake, as previously observed in rainbow trout fed high oregano oil levels (36). Second, while essential oils can act as antioxidants at low levels, they may exert pro-oxidant or cytotoxic effects at high concentrations, thereby increasing metabolic stress or damaging the intestinal epithelium (37). This is supported by Reverter et al. (38), who reported biphasic effects of plant-derived bioactives in aquafeeds. Third, the cumulative intake of the carrier solvent, propylene glycol—especially at 240 mL/kg HEO (~36 g/kg diet)—may have exerted subclinical toxicity or impaired digestive enzyme activity, although this was not directly measured. Together, these findings highlight the importance of determining optimal inclusion levels. Based on our regression modeling and observed trends, we recommend an upper inclusion limit of 60 mL kg^−1^, beyond which HEOs may exert counterproductive effects.

The immune-enhancing effects observed in fish fed 30 and 60 mL/kg HEOs—evident through elevated lysozyme activity, phagocytic rate, and IgM levels—suggest stimulation of both innate and adaptive immune pathways (34). These effects are likely mediated by carvacrol and thymol, which have been shown to activate macrophages, promote phagocytosis, and modulate cytokine release. Carvacrol, in particular, enhances the expression of pattern recognition receptors and increases microbial clearance. Furthermore, the significant upregulation of antioxidant enzymes (SOD and CAT) in these groups indicates improved oxidative defense, potentially driven by the phenolic structure of thymol and limonene, which facilitates neutralization of reactive oxygen species (ROS). The lack of significant changes in MDA levels supports the notion that oxidative stress was well controlled across treatments. These findings demonstrate that moderate doses of HEOs promote immune readiness and redox homeostasis, contributing to improved disease resilience (9, 34).

The Studies of functional feed additives on aquatic species generally use the transcription of growth, immunological, and related genes to discover the genetic mechanism of action (39). In Nile Tilapia fed 0.4 and 0.5% OVLE, Hsp70 was downregulated, which is important for fish health (40). According to the study, HSP70 expression was dramatically reduced in fish fed 30- and 60-ml HEOs/kg−1 food. This research supports prior findings (34). Fish fed 1 g/kg of oregano essential oil showed a considerable reduction, with the lowest level.

Glycoprotein indicators such as IL-1β, IL-8, IL-10, and IgM regulate fish immune response, represent innate immunity, and alter viral disease response (41, 42). Consistent with Giri et al. (43), IL-1β, a pro-inflammatory cytokine, activates lymphocytes and enhances the release of other cytokines like TNF-ɑ. Our investigation found that fish fed 30- and 60-ml HEOs/kg−1 had considerably lower IL-1β and TNF-*α* mRNA levels than other experimental groups. Similarly El-Bab et al. (9), results indicate that fish given 0.2 and 0.4% OVLE had higher levels of IL-1β, whereas those fed 0.4 and 0.5% OVLE had higher levels of IL-8. It supported these findings (44). Who examined how thyme and *Nigella sativa* affect these Nile tilapia genes through food. Additionally, NS dietary additives dramatically increased IL-1β, IL-8, and IgM expression (44). Abd El-Hamid et al. (45) increased CNE levels led to greater IL-1β, IL-8, TNFα, and IL-10 gene expression compared to the control group. Zebrafish fed encapsulated cinnamaldehyde demonstrated increased IL-1β, TNFα, and interferon-gamma expression levels (46).

Occludin and ZO-1 help tighten junctions. Occludin is the major structural and functional component of tight junction proteins that preserves junction integrity (47). As a tight junction-associated adaptor protein, ZO-1 links occludin/claudin and actin cytoskeleton to maintain tight junction stability and function (47). The study found that fish fed 30- and 60-ml HEOs/kg−1 diets had considerably higher ZO-1 and occludin expression levels than other groups. The 60 mL diet had the highest mRNA level. According to an earlier study, Oxazolone inhibited ZO-1 and occludin transcription in zebrafish (48). The GH/IGF axis controls somatic growth. One transmembrane receptor regulates the (GHRs) in teleost fish, which have two paralogous versions, GHR-I and GHR-II, with complementary roles (49). Higher fish weights are related to larger IGF-I mRNA expression in fish of the same species, age, and rearing conditions under optimal feeding regimens (35). In our investigation, fish fed 30- and 60-ml HEOs/kg−1 exhibited significantly greater GHr and IGF-I mRNA levels in hepatic tilapia compared to other groups. Our findings contradict recent research showing higher IGF-I expression in (*O. mossambicus*) (50). Previous Nile tilapia research found a positive association between liver IGF-1 protein, mRNA levels, body weight increase, and SGR (51). Polyphenols can increase hunger and food consumption by improving the digestive enzymes, DNA, RNA, GH, IGF-1 creation, and ghrelin (GHRL) expression. Additionally, improving the microbiota in the intestines can improve metabolic functions and growth, contributing to general health and development (35).

The dose–response pattern was nonlinear, with performance improvements plateauing between 30 and 60 mL/kg, followed by a decline at higher doses. This was confirmed using broken-line and quadratic regression models, which estimated an optimal HEO dose of ~53–56 mL/kg for growth and gene expression endpoints. The decline in performance at higher doses may be due to bioactive compound overload or excessive carrier (propylene glycol) intake.

One limitation of the current study is the relatively low number of replicates (three tanks per treatment), which may limit the detection of subtle effects. However, *post hoc* power analysis indicated sufficient power (>80%) to detect medium-to-large effects observed in growth performance and gene expression. Future studies with increased replication and factorial designs are encouraged to confirm and refine these findings.

## 5 Conclusion

The present study demonstrates that dietary inclusion of herbal essential oils (HEOs) at 30–60 mL kg^−1^ diet significantly improves growth performance, feed utilization, antioxidant defense, immune response, and intestinal morphology in Nile tilapia. These benefits are associated with upregulation of growth- and immunity-related genes and improved intestinal histoarchitecture. However, higher inclusion levels (≥120 mL kg^−1^) were detrimental, suggesting a threshold beyond which HEOs may exert adverse effects. Therefore, 30–60 mL kg^−1^ appears to be the optimal inclusion range under current conditions. Future research should validate these findings under commercial aquaculture conditions, considering different life stages and feeding regimes.

## Data Availability

The original contributions presented in the study are included in the article/Supplementary material, further inquiries can be directed to the corresponding author.
