# Supplementary material for: Herbal essential oils improve growth, antioxidant response, and gene expression in Nile Tilapia fingerlings
Source: Front Vet Sci. 2025 Sep 10;12:1620632. doi: 10.3389/fvets.2025.1620632 (PMC12458646; doi:10.3389/fvets.2025.1620632)
Supplement: Supplementary file 1 [file Data_Sheet_1.PDF]

Supplementary Table S1 - GC-MS Composition of HEO Blend

Component	Concentration (g/L)	Chemical Class
Carvacrol	45	Phenol
Thymol	39.2	Phenol
Oregano oil	45	Essential oil
1,8-Cineole	16	Monoterpene oxide
Î±-Pinene	4.6	Monoterpene
Î^{2}-Pinene	2.6	Monoterpene
Limonene	3	Monoterpene
Propylene Glycol	150	Carrier solvent

The chemical makeup and concentrations of the herbal essential oil blend (TRI-VIR™). Include it as a supplemental table in your submission package.

Supplementary Table S2: Goblet Cell Density (expressed as cells per 100 µm of villus length) across intestinal regions and treatments:

Treatment (mL/kg)	Anterior Intestine (cells/100 µm)	Middle Intestine (cells/100 µm)	Posterior Intestine (cells/100 µm)
0	5.8 ± 0.4[a]	5.4 ± 0.3[a]	5.1 ± 0.2[a]
30	7.3 ± 0.3[b]	6.9 ± 0.2[b]	6.5 ± 0.3[b]
60	8.1 ± 0.3[b]	7.9 ± 0.2[b]	7.2 ± 0.3[b]
120	6.2 ± 0.3[a]	6.0 ± 0.2[a]	5.7 ± 0.2[a]
240	5.7 ± 0.2[a]	5.5 ± 0.2[a]	5.2 ± 0.2[a]

Values are presented as mean ± SEM (n = 15 observations per treatment). Different superscripts within a column indicate significant differences ($P < 0.05$).
